# Zebrafish as an Innovative Tool for Epilepsy Modeling: State of the Art and Potential Future Directions

**DOI:** 10.3390/ijms24097702

**Published:** 2023-04-22

**Authors:** Marta D’Amora, Alessandro Galgani, Maria Marchese, Francesco Tantussi, Ugo Faraguna, Francesco De Angelis, Filippo Sean Giorgi

**Affiliations:** 1 Istituto Italiano di Tecnologia, 16163 Genova, Italy; 2 Department of Biology, University of Pisa, 56125 Pisa, Italy; 3 Department of Translational Research and of New Surgical and Medical Technologies, University of Pisa, 56126 Pisa, Italy; 4 Molecular Medicine and Neurobiology-ZebraLab, IRCCS Fondazione Stella Maris, 56128 Pisa, Italy; 5 Department of Developmental Neuroscience, IRCCS Fondazione Stella Maris, 56128 Pisa, Italy

**Keywords:** zebrafish, epilepsy, seizures, pentylenetetrazol, kainic acid, pilocarpine

## Abstract

This article discusses the potential of Zebrafish (ZF) (Danio Rerio), as a model for epilepsy research. Epilepsy is a neurological disorder affecting both children and adults, and many aspects of this disease are still poorly understood. In vivo and in vitro models derived from rodents are the most widely used for studying both epilepsy pathophysiology and novel drug treatments. However, researchers have recently obtained several valuable insights into these two fields of investigation by studying ZF. Despite the relatively simple brain structure of these animals, researchers can collect large amounts of data in a much shorter period and at lower costs compared to classical rodent models. This is particularly useful when a large number of candidate antiseizure drugs need to be screened, and ethical issues are minimized. In ZF, seizures have been induced through a variety of chemoconvulsants, primarily pentylenetetrazol (PTZ), kainic acid (KA), and pilocarpine. Furthermore, ZF can be easily genetically modified to test specific aspects of monogenic forms of human epilepsy, as well as to discover potential convulsive phenotypes in monogenic mutants. The article reports on the state-of-the-art and potential new fields of application of ZF research, including its potential role in revealing epileptogenic mechanisms, rather than merely assessing iatrogenic acute seizure modulation.

## 1. Introduction

Epilepsy is a common neurological disorder that affects both children and adults, yet many aspects of this disease remain poorly understood. The purpose of this review is to provide an overview of the current state of research on ZF with a focus on its potential as a model for epilepsy research. In fact, while the burden of epilepsy on patients and their communities highlights the need for a better understanding of its pathophysiology and the development of treatments that can cure seizures, the use of classical in vivo and in vitro models derived from rodents presents significant limitations. Despite the relatively simple brain structure of ZF, researchers can obtain several relevant insights into epilepsy pathophysiology and treatment (when keeping adequately into account the potential limitations and risk of overinterpreting findings obtained in this animal species), as well as a large amount of data in a much shorter period and at lower costs compared to classical rodent models. This is particularly useful when a large number of candidate antiseizure drugs (ASDs) need to be screened, and ethical issues are also minimized. The utility of Zebrafish in modeling central nervous system (CNS) disorders is evidenced by the large number of papers published in highly authoritative scientific journals over the last decade, in which Zebrafish were used to model aspects related to complex central nervous system disorders, including Parkinson’s and Alzheimer’s Disease. An overview of the different seizure models and assessment tools used in ZF will be provided, together with their potential role in clarifying different aspects of epilepsy research.

## 2. Methods

**Figure 1:**
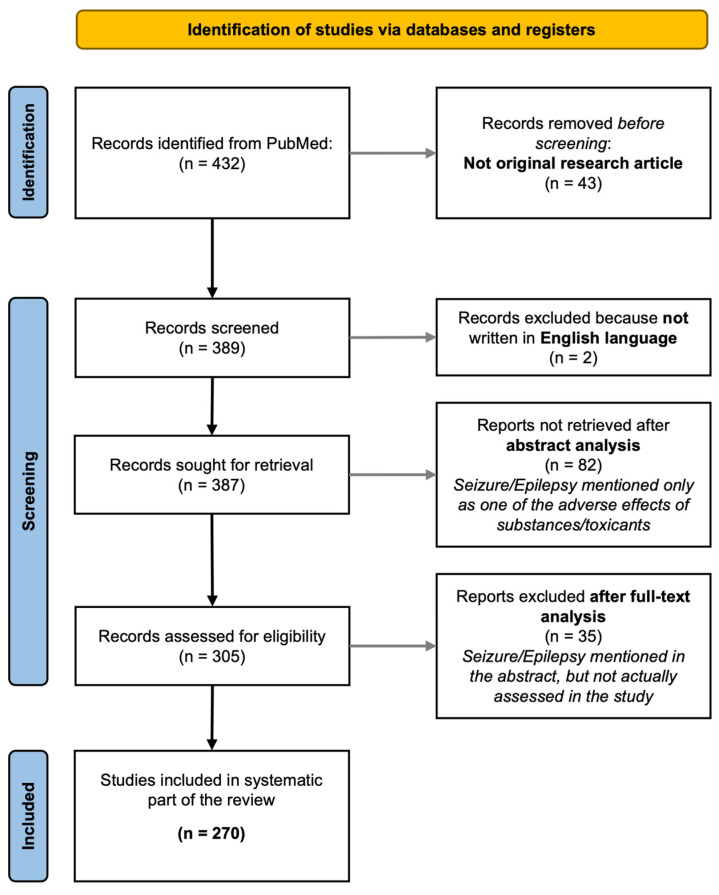
The workflow of the literature search is reported in the flowchart. The search was performed on pubmed.ncbi.nlm.nih.gov using the keywords reported in the Section 2.

This is a narrative review. However, we started from material collected by using the pubmed.ncbi.nlm.nih.gov search platform to perform a literature search. In particular, we searched for papers describing seizures in ZF, using the keywords “seizures” and/or “epilepsy”, and “zebrafish”. We included only original articles written in English, dealing with the use of ZF to assess, at least among other parameters, seizures (papers describing seizures as side effects of compounds tested on ZF were not included). Figure 1 shows the flow chart of the literature search. The review of collected studies was performed by four of the authors (MDA; AG; MM; and FSG). The review was not registered, and no review protocol has been prepared.

## 3. The Role of Animal Models in Epilepsy Research

Epilepsy is a brain disorder affecting more than 50 million persons worldwide [1]. It is featured by the recurrence of seizures which can be defined as the effect of abnormal and/or excessive discharge of cortical neurons. The term “epilepsy” may be rather misleading, and it should be replaced by “epilepsies” since similar types of seizures can be the results of different genetic, “idiopathic”, structural, focal, or generalized alterations occurring in the brain.

Seizures can manifest with different phenotypes depending on the brain areas most affected by them; however, they often involve motor areas causing motor manifestations, such as clonic, tonic, and tonic–clonic seizures. Many aspects of the pathogenesis of different types of seizures have been clarified in the last decades, and a variety of epilepsy syndromes have been described [2,3,4,5]. In adults, most of the seizures are related to structural brain alterations (e.g., post-traumatic, poststroke, or temporal lobe epilepsy due to hippocampal sclerosis), while in children, congenital genetic epilepsy syndromes are quite frequent; the dramatic advances in genetics have allowed for the identification of a large number of these syndromes in the last decades. In parallel, advanced neuroimaging tools, together with sophisticated electroencephalogram (EEG) approaches have also allowed for the identification of epilepsy causes in patients previously classified as affected by “cryptogenic” seizures.

Today, a variety of ASDs are available, which, however, interestingly have been developed in most cases after the serendipitous discovery of their anticonvulsant effect rather than by designing target-specific compounds [6,7]. Yet, despite such a relative abundance of ASDs, still today approx. 30% of epilepsy patients have uncontrolled seizures, i.e., are ”pharmaco-resistant” [1]. This causes a dramatic burden, not only for the patients themselves but, in terms of healthcare costs, also for their countries’ welfare systems. Thus, a strong effort has been dedicated in the last decades by public and private research funding agencies, and pharmaceutical companies, to fund studies investigating new aspects of the pathophysiology of different epilepsy syndromes, as well as to developing new ASDs.

Experimental models have been crucial for helping to clarify several aspects related to epilepsy pathophysiology and treatment. For both these purposes, rodent models have been the most extensively used in the last decades. For ASD screening, starting from as early as in the 1930s it was developed the maximal electroshock (MES) model [8], in which tonic–clonic seizures were induced by cornea- or ear-applied electrical shock in rats, followed a few years later by the introduction of routine ASD screening, also of the pentylenetetrazol model in rats and mice [9,10]. The former was considered a model of secondarily generalized seizures, while the PTZ model has been considered a model of primarily generalized seizures, either nonconvulsive (or absence-like) or convulsive ones, depending on the dose and route of administration of the chemoconvulsant [11,12]. These models have been extensively used thereafter to provide a first screening of candidate ASDs, together with other rodent models which were added more recently (for a review, see [12]). Among those, some models aim to reproduce at least some aspects of limbic epilepsy, i.e., epilepsy featuring seizures starting from the limbic structures, which is very common in humans [13]. With this respect, the most popular ones are the KA and pilocarpine models in mice and rats, which are based on the systemic administration of these chemoconvulsants that cause seizures starting from the hippocampus–limbic system [14,15,16,17,18,19]. In mice and rats, KA and pilocarpine induce prolonged seizures (i.e., status epilepticus, SE), which are associated with neuronal damage in several parts of the limbic system itself and, after a varying degree of time, also induce the occurrence of spontaneous recurrent seizures (SRS) (e.g., see [20]; reviewed in [21]). Due to this delayed effect, both have been used as models of “epileptogenesis”, and used to test the antiepileptogenic potential of ASDs, when administered during the interval period between the SE (first hit) and SRS occurrence (e.g., see [20]; reviewed in [21]). Despite their potential appeal in simulating the epileptogenesis process, some aspects of these “two-hit” models have been challenged as not very “realistic” ones (see below). Limbic epileptogenesis has also been reproduced by “kindling” procedures, i.e., by repeatedly administering low-intensity stimuli in specific limbic areas (e.g., hippocampus or amygdala), which eventually lead to the appearance of seizures, that can be evoked by the same stimulus even after a delayed time interval [22,23], or even lead to spontaneous seizure occurrence [24].

The same abovementioned models of limbic seizures/epileptogenesis have been applied in the last decades to rodent pups at different postnatal ages, to investigate aspects related to developmental epilepsy; interestingly, in general, the doses of convulsant and stimulation intensities needed to induce SE or kindling, respectively, are much lower than in adult rodents [16,17,25,26,27,28,29,30] confirming the higher incidence of seizures observed in the pediatric population, compared with middle-aged one, also in humans.

The rodent models listed thus far bear many limitations. For instance, despite being accepted as models of epileptogenesis, some aspects of the abovementioned two-hit models (i.e., SE induced by KA or pilocarpine, followed by brain damage causing SRS) have been put in discussion as poorly applicable to the human condition, as previous iatrogenic strong insults, similar to those occurring after the KA or pilocarpine administration, are only rarely occurring in patients’ history. Furthermore, spontaneous recurring seizures occur in a minority of animals, and they can be often identified only with chronic EEG recording, which is technically challenging for the researcher and stressful for the animal. In any case, those rodent models which are mostly used for ASD screening (PTZ, MES), and in which seizures are induced acutely and evaluated only behaviorally, are becoming increasingly difficult to perform due to the high number of animals required, raising ethical issues. Furthermore, these studies are expensive, since they need a significant investment in animal care, staff, and facilities by the funding agencies/institutions involved.

**Figure 2:**
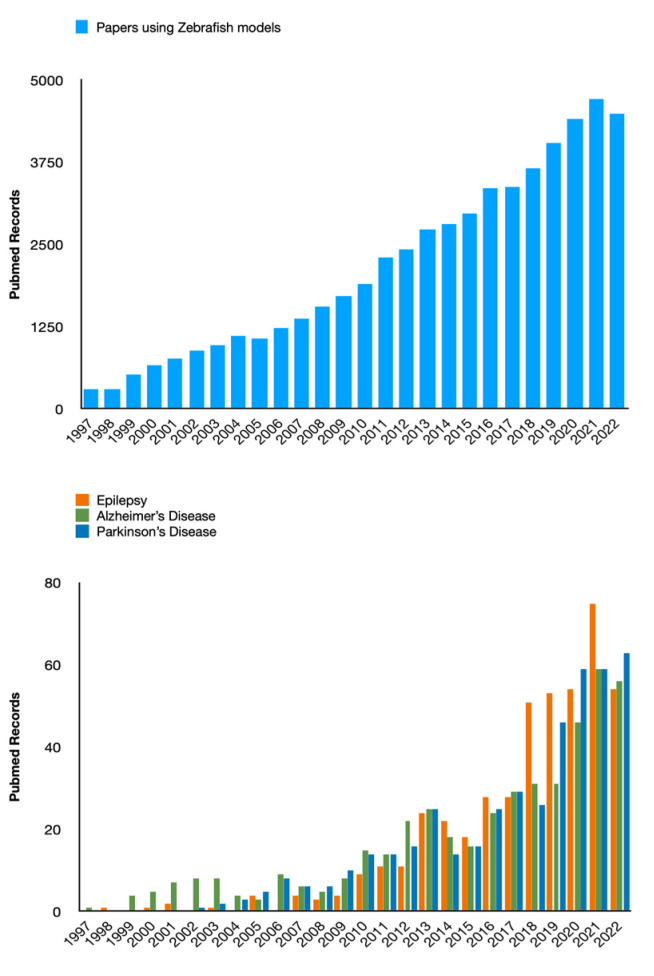
The histograms report the amount of papers published in PubMed-indexed journals in the last 25 years. The total count has been continuously growing, both considering ZF as a model (top graph) and its use in the field of neurological disorders (bottom graph). (Please note that this figure has been produced only for demonstrative purposes and the data shown are unrelated to the methodology and the collected results of the systematic part of this review paper).

ZF has been used for the first time as a model of seizures in 2005 by Baraban’s group [31]. In that seminal paper, the authors detailed the PTZ model in ZF larvae, and, at the same time, detailed the behavioral and extracellular electrical activity, as well as the expression of *c-fos* as an index of neuronal activation. This study set the scene for the future development of the ZF models of epilepsy. Indeed, after initial skepticism by some researchers in the field, noted by Baraban himself, in a recent paper [32], an increasing number of papers have been published thereafter by several labs around the world (Figure 2).

## 4. The Use of ZF as Epilepsy Models: State of the Art

### 4.1. ZF General Features Making It Fit for Studying Epilepsy

ZF belongs to the family of barbs, the order of Cypriniformes, the class of Osteichthyes, the Subphylum Vertebrates, and the Phylum of chordates, which originated from South Asia. It is a small teleost fish, whose embryonic development is very fast, and the whole plan of body development is already defined as early as 24 h postfertilization (hpf). This rapid development is comparable to that of a human embryo in the first three months of intrauterine life [33]. The ZF as a vertebrate model organism has been used in numerous studies of various human diseases dating back to the 1980s [34], including cancer, cardiovascular disease, and metabolic disorders. In the last decade, the number of scientific papers in which ZF have been employed to study and model neurological disorders, including neurodegenerative ones (such as Alzheimer’s Disease, and Parkinson’s disease) and epilepsy increased dramatically, as shown in Figure 2. The main reasons for this are likely represented by specific peculiarities of these vertebrates, including short life span and rapid generation time, genetic similarity to humans, and reduced ethical constraints compared to mammals (at least concerning larval stages) [35]. Similar to mice and fruit flies, ZF can be used for genetic analysis. Moreover, the external development of ZF larvae and their optical transparency make their CNS easily accessible for experimental manipulation. The use of a combination of genetics, embryology, and state-of-the-art optical techniques makes the ZF a unique model organism to study neurogenesis and epilepsy, showing numerous advantages over its murine counterpart, allowing faster results, and reducing the costs for pharmacological testing.

In the last decade, ZF embryos/larvae have emerged not only as organisms useful for disease modeling but also as alternative preclinical models for drug discovery and screening, since they enable rapid evaluation of the efficiency and potential toxicity of new drug candidates [36,37,38]. ZF presents a high physiological similarity with humans and suitability for the replacement, reduction, and refinement (3Rs), hence is potentially able to mimic human responses [35,36]. In comparison with cultured cells, ZF can give information on drug absorption, distribution, metabolism, excretion, and toxicity (ADME) [37]. A study in ZF allows us to understand the effects of drugs in a whole and complex system, at cellular, tissue, and organ levels, and performing screening in a broad range of phenotypes. In addition, thanks to their high fecundity rate and small size, tests can be performed in up to 96-well plates, reducing the amount of drug required for the test (see below).

All these features make the use of ZF an ideal candidate for the in vivo high-throughput toxicological screening of drugs with higher accuracy, lower costs, lower duration, and lower ethical impact compared with mammalian models. This allows not only a drug phenotype-based evaluation but also the possibility of repurposing existing drugs for personalized treatments [38].

It is worth being mentioned that, even though simplified, in the CNS of ZF several subcortical and cortical structures are phylogenetically old enough to be conserved and preserved in terms of the cellular features of the neurons which constitute them, and their main connections. The ZF brain, like that of any vertebrate, is subdivided into three main sections: forebrain, midbrain, and hindbrain/spinal cord. Further subdivisions occur early on and generate specialized structures within these domains [39]. From the embryonic structures of the forebrain, midbrain, and hindbrain, cells differentiate and form adult brain structures, such as the pallium, subpallium, thalamus, and cerebellum [40]. Therefore, the majority of studies conducted so far focus on neurogenesis in the embryonic stages. However, recent studies have shown that the mature ZF brain may also serve as a valuable tool to study adult neurogenesis [41]. There are differences between the ZF brain and the human brain; for instance, the telencephalon does not develop into a layered cortex. However, there are also structures highly homologous between the two species, such as the habenula, the layered structure of the cerebellum, the hypothalamus, and its neuroendocrine systems. In addition, most of the neurotransmitters and the neuropeptidergic system [42] are highly similar between the two brains.

Over the past decade, ZF received an enormous boost as an organism fit for modeling specific diseases thanks to the advent of novel tools allowing targeted mutation in a relatively easy and rapid manner, including new, efficient strategies of gene editing, such as the transcription activator-like effector nucleases (TALEN) system ([43], and, even more recently, clustered regularly interspaced short palindromic repeats (CRISPRs)/cas9 system [44]. The other advantage of using ZF for post-genome neurobiology is that there is a large collection of gene expression patterns, as well as available mutant and transgenic strains. All these aspects make ZF a powerful model organism for forward and reverse genetics [45]. With its transparency and small brain, the ZF is an ideal animal model for monitoring brain activity with calcium imaging [46]. The introduction of two-photon microscopy to calcium imaging in ZF allowed the optical sectioning of brain tissue noninvasively. While the optical monitoring of brain activity was transforming neurophysiology, another revolution in neuroscience was taking place: optogenetics, a novel technique that uses light to activate or silence genetically altered neurons [47]. Recently, ZF became a valuable model also for neurobehavioral studies since larvae display several, quantifiable behavioral phenotypes that can be monitored by video-tracking devices. Therefore, larvae (until five days postfertilization, dpf) tend to be chosen for ZF studies, focusing on a very small-time window in the ZF life span. Hence, using larval neurological disease models should consider the fact that the neural system is incompletely developed, which may limit the investigable aspects of some neurological disorders. Moreover, larvae exhibit dynamic changes through their ontogenesis, while adults are physiologically more stable and have a full range of complex and integrated brain functions, showing that the benefits of adult ZF are often undervalued [48].

It has been demonstrated by using behavioral, molecular, pharmacological, and electrophysiological analysis, that ZF experiences seizures reproducing many of the crucial aspects of mammalian seizures [49], making them an excellent tool for studying epilepsy. In terms of seizure evaluation, ZF larvae have been shown to exhibit a range of motor behaviors reminiscent of those seen in humans with epilepsy, which includes body shaking and changes in swimming patterns, which are easily recognized and staged (see below). Finally, as said, ZF has a rapid life cycle, allowing for the study of multiple generations in a relatively short period. This allows for the long-term effects of seizures in a single subject, as well as the potential development of epilepsy to be inherited across generations, to be quickly examined. The minuscule size of larvae currently prevents the simultaneous recording of behavior and EEG, though ZF offers several unique advantages in epilepsy research. For example, ZF membranes are permeable to drugs placed in the bathing medium. The ease with which drugs can be delivered to freely behaving ZF and the sensitivity of locomotion and electrophysiology assays for seizure activity have favored novel moderate- to high-throughput platforms for the discovery of innovative ASDs that can be transferred to rodents and then potentially tested in patients [50].

#### Differences between Larval and Adult Zebrafish in Epilepsy Studies

Thanks to all of the features described above, ZF larvae are generally employed to study epilepsy, while adult characteristics are still underestimated. ZF larvae and adults present many differences in terms of pathophysiological and pharmacological aspects that should be taken into account during epilepsy investigations.

First, as already described, adults possess a fully developed brain, with a complex structure including the blood-brain barrier (BBB) [48]. In fact, during the growth from larva to adult stage (>90 days), the peripheral and central nervous systems are subjected to maturation as well as all the other organs, until reaching physiological stability [51].

The ZF BBB presents complex organization and function that are similar to the mammal’s [52]. Its endothelial cells possess tight junctions that are responsible for the size-dependent permeability of substances. The BBB formation starts at 3 dpf and is almost completed at 14 dpf. Since the ZF BBB functionally and structurally matures and changes during the larva/juvenile stages, it should be considered that the implications in epilepsy and the delivery/effects of ASDs can be different between larvae and adults. This is extremely important considering that the disruption of BBB is involved with the impact, origin, and treatment of seizures [53].

As above described, neurogenesis in larvae and adults presents several differences, with two phases in the larval period and an intensive process in the adults that involve all the brain area, and different neuronal precursor expressions between the two stages. In addition, as for mammals, seizures induced in adults by PTZ stimulate neurogenesis in a specific region of the dorsal telencephalon [54]. This suggests that the study of seizures in adult ZF may help in understanding the role of neurogenesis on human epileptogenesis.

Another difference between the larvae and adults that can be relevant in epilepsy research is represented by the different uptake and delivery of compounds or drugs from the external medium. On one side, the larvae, until 48/72 hpf, are surrounded by a protective barrier, named chorion, that presents small pores, enabling the compounds/drugs to be internalized by passive diffusion. After 72 hpf, the larvae can uptake the compounds by ingestion through the gut, gill, and skin. During drug exposure, a high number of embryos/larvae are generally placed in a 24-well plate and treated simultaneously to different doses of compounds/drugs diluted in an embryo medium. On the other hand, the adults are treated with compounds/drugs by water dispersion in the tanks. ZF adults absorb the compounds/drugs through the gut, gill, and skin. However, adults possess a fully developed BBB, and consequently, the different compounds/drugs need to cross this protective barrier to reach their specific CNS targets. It is observed that the ability of drugs to penetrate or not the BBB in zebrafish is similar to the one of mammals. For instance, haloperidol, scopolamine, and diphenhydramine are significantly absorbed by the ZF as they can easily cross the mammalian BBB, while the desloratadine and scopolamine N-butyl bromide did not cross the BBB [52].

Even if the drug exposure in the multiwell plate for embryos and in the water tank for ZF adults is an easy and fast tool, this route of administration does not give any information regarding the amount of drug that is uptaken from the zebrafish. Consequently, this approach is inappropriate in the case of drugs that are poorly soluble in water, for instance in the case of primidone. In ZF adults the level of drugs in the CNS can be evaluated, after extraction of the brain. However, this quantification requires sacrificing the animals and it is not easily applicable to the larvae. To overcome these limitations, other routes of administration can be used both in ZF larvae and adults. In larvae, the drugs can be administered by microinjection, while in the adults the intraperitoneal and oral ones are the most commonly employed.

Even if the impact of sex in epilepsy is not clear, this parameter needs to be taken into account during the different epilepsy investigations since it presents differences between larvae and zebrafish. In humans and rodents, sex hormones affect ASDs metabolism, epilepsy development, and seizure susceptibility [48,55,56]. In ZF larvae at 3 dpf, many genes are insufficiently expressed, making sex determination difficult. Thus, sex differences can only be considered in adults. These changes include diverse functional or proliferative activities in some brain regions in ZF females and males [57,58] and different expression patterns of neuronal genes [59]. Despite this, sex is a parameter that is generally poorly considered during the ZF epilepsy assessments. In fact, only two studies investigated the possible different behavior and seizure states in males and females reporting no changes between the two sexes [60,61].

### 4.2. Use of Chemoconvulsants in ZF

In ZF, seizures have been induced through a variety of chemoconvulsants, among which, however, PTZ is by far the one that has been used by most groups, starting from the original paper by Baraban et al. (2005) [31]. Pentylenetetrazol is a gamma-aminobutyric acid (GABA)-receptor antagonist, which has been tested extensively in a variety of animal species, especially mice and rats. In rodents, the dose of PTZ required for the induction of seizures can vary depending on the strain, sex, and age of the animals, as well as the route of administration. Typically, for mice, the dose range of PTZ is between 50 and 100 mg/kg, while a dose of 60 mg/kg is a commonly used dose for inducing seizures in adult rats; in both models, PTZ is administered by a single intraperitoneal (i.p.) injection. PTZ has been classically used in rodents as a tool to screen ASDs rather than to reproduce and explore the pathophysiology of epilepsy. This is particularly true in light of the different types of seizures which are reproduced at increasing doses of the drug [62], with doses as low as 30 mg/kg inducing absence-like seizures, and doses above 100 mg/kg to reproduce generalized tonic–clonic seizures. In detail, the most used protocols for PTZ use in mice during ASD screening profit from its s.c. administration at a dose inducing clonic seizure of at least 5 s in at least 97% of animals within 30 min (usually around 80 mg/kg), as the “threshold” test for ASD toward generalized nonconvulsive (myoclonic or absence) seizures [11].

In ZF, PTZ induces a quite stereotyped sequence of motor behavior, which was initially described by Baraban and colleagues (2005), and which is detailed in Section 4.1. After the first description of seizure motor staging through visual inspection, then, automatic and semiautomatic video tools became available and are currently widely used (See Section 4.1). PTZ has been, ever since, the one, by far, most used chemoconvulsant used in ZF. It is considered, similar to its interpretation in rodents, as a model of generalized seizures and especially absence-like and generalized tonic–clonic seizures [31,63].

Another chemoconvulsant that has been used in ZF is represented by KA. As said in Section 3, KA is, by far, the most used chemoconvulsant for inducing limbic seizures and SE in rodents at different ages. In ZF, most of the few studies on KA have been performed in adult animals, where it was administered intraperitoneally to induce seizure-like behavior with clonic convulsions occurring in all ZF treated with 6 mg/kg and becoming prolonged up to frank SE at the dosage of 8 mg/kg [64]. Interestingly, the latter doses are close to those used in rodents [14]. Furthermore, KA is the only model of seizures claimed to reproduce SE in ZF, thus far, and it is worth noting that reproducing SE is also the main aim by which KA is used in rodents. Indeed, a specific parallel with the adult rodent KA-induced SE model, also in terms of biochemical markers of SE, was done by Mussulini et al. in an ad hoc designed study [65]. In larval ZF, a recent interesting protocol consisted of infusing low KA doses in the common cardinal veins in the pericardium at 3 dpf. In the latter model, the behavioral and EEG effects of KA were well detailed as well as the antiepileptic effects of different ASD and the neurochemical effects of seizures [66]; however, this model is quite challenging from a methodological point of view. It is worth mentioning that in ZF, larvae KA has been shown to be able to induce reproducibly convulsive activity also after solubilization in fish water [67], even though its behavioral effects were not detailed in the study employing this route of administration. Interestingly, in one previous study in which KA was bath applied to ZF larvae, clear convulsive seizures resembling those described in adult ZF by Alfaro et al. were not observed [66], even though a much lower concentration of KA in fish water (50 µM) was able to induce electrographic activity by Kim et al. in 2011 [68].

Pilocarpine is a nonselective muscarinic agonist with a high affinity for CNS muscarinic receptors, that is commonly used to induce seizures in different animal models, including rodents, for the study of epilepsy. Pilocarpine has been used only very recently in adult ZF, even though, with different doses and protocols of administration [69,70,71], uses a score similar to the one used for KA [72]. Interestingly, in these studies, the chronic effects of pilocarpine-induced seizures were assessed with repeated administration inducing delayed reduced concentrations of GABA, an increase in inflammatory markers [69], a single administration of pilocarpine inducing subtle alterations of some molecular synaptic and neuro proliferation markers, and repeated administration of pilocarpine followed by one PTZ shot induced significant cognitive and neurotransmitter alterations [70]. Pilocarpine had also been used in ZF larvae [73,74]; interestingly, Winter et al. (2017) showed that, in 4 dpf ZF larvae, the activation after exposure to up to 1.25 mM pilocarpine for 30 min is quite focused on telencephalon (as assessed by functional neuroimaging, using genetically-encoded Ca^2+^ sensors, which have a high spatiotemporal resolution) and less wide than that of PTZ, 4-AP, and strychnine, proposing this chemoconvulsant as a promising one in larvae.

Another GABA-A receptor antagonist that has been used commonly in epilepsy models, bicuculline, has been used sporadically to induce seizures in ZF larvae, though the behavioral manifestations have not been described in detail thus far, nor has it been used as a model to screen ASDs [67].

Finally, it is worth mentioning also other chemoconvulsants which have been used sporadically to induce seizures in ZF. These include N-methyl-D-aspartate (NMDA), a glutamatergic receptor agonist, administrated either by i.p. or by immersion, causing reproducible severe seizures in adults [75]; or ethyl ketopentenoate, a glutamate decarboxylase inhibitor, which induces pharmacoresistant seizures in ZF larvae [76,77]. Accordingly, another glutamate decarboxylase inhibitor, allyglycine, was shown to induce frequent seizures in ZF larvae which are prevented by a variety of ASDs [78]. Finally, in line with the proconvulsant effect of PTZ, recently, different authors assessed the effect of another well-known GABA-A receptor antagonist, picrotoxin, larval ZF [67,79,80] or adult ZF [59], confirming a seizure behavior effect similar to PTZ itself.

### 4.3. ZF as a Tool for Assessing Genetic Causes of Epilepsy

Apart from chemoconvulsant-induced seizures, spontaneous seizure-like activity occurring in ZF with specific gene mutations has been described as well. In the last decade, several novel ZF lines have been developed by using different genetic-engineering approaches, including morpholinos (MOs), zinc-finger nuclease (ZFN), TALEN, RNA interference, or CRISPR silencing (Table 1).

One of the most frequently used ZF epilepsy models is the Dravet Syndrome model, characterized by a mutation in the *SCN1A* gene, encoding for a voltage-gated sodium channel. Different ZF lines with *Scn1Lab* (sodium voltage-gated channel alpha subunit 1) loss have been developed and investigated at the behavioral level [81,82,83,84]. The *Scn1Lab* mutants exhibited increased locomotor activity and seizures, with behavior classifiable as Stage III [82]. Remarkably, this activity was similar to the one described in larvae treated with PTZ by Baraban et al. [31]. In addition, a decrease in seizures in *Scn1Lab* larvae was observed after treatment with certain drugs, including stiripentol, potassium bromide, diazepam, and valproate [82], while the exposure to other ASDs did not cause any effect on the seizure phenotype [82]. Moreover, larvae exhibited an increase in the number of movements between 3 and 5 dpf, with abnormal hyperactive locomotor activity in this temporal window [84], including tremors. Finally, these mutants were employed to identify drugs able to suppress seizures by a screening of thousands of compounds (1012) [83]. Among the analyzed compounds, 20 of those were able to induce a decrease or total disappearance of convulsions [83].

Other mutations in genes encoding for a potassium channel are associated with a complex phenotype including epilepsy, ataxia, sensorineural deafness, and renal tubulopathy (EAST/SeSAME syndrome) [85,86]. In particular, *kcnj10a* larvae, characterized by a mutation in the inward-rectifying potassium channel Kir4.1, displayed alterations in their movements both at early stages with contractions and at 5 dpf with erratic posture [85]. In addition, larvae presented seizure events that were unresponsive to the treatment with diazepam but suppressed by exposure to pentobarbitone [86]. Furthermore, mutations in another family of channels, the KCNQ3, led to benign familial neonatal convulsions (BFNC) [87]. Morphant larvae treated with 25–200 µM of linopirdine (LPD) exhibited a significant increase in swimming activity. In addition, exposure to a low dose of LPD caused behaviors classifiable as Stage I, while treatment with a high concentration of drugs led to a locomotor activity similar to Stage III.

Among the other ZF epilepsy models they are worth being mentioned, in particular, the seizure-like behavior described as *mind bomb* (mind bomb E3 ubiquitin protein), and *lg1a* (leucine-rich glioma-inactivated 1a) mutants. These mutant ZF show an increase in the number of movements as well as behavioral manifestation closely reminiscent of similar PTZ-induced seizures [88]. In particular, *mimb momb* larvae are mutants of the notch signaling pathway, while the *lg1a* mutants bear an alteration in a secreted protein involved in protein–protein interactions. This latter can cause autosomal dominant lateral temporal lobe epilepsy (ADLTE) or autosomal dominant partial epilepsy with auditory auras (ADPEAF). On one side, the total loss of *lg1a* in ZF larvae induced several abnormalities during the development, such as pericardial edema, malformations in the tail, and smaller eyes in comparison to control animals. These larvae displayed hyperactivity with behaviors classifiable as Stage I and II and characterized by myoclonic-like jerks and whirlpool swimming [89]. On the other side, a partial loss of *lg1a* did not induce a seizure phenotype but caused a higher sensitivity to convulsant drugs. In particular, treatment of larvae with 2.5 mM of PTZ led to hyperactivity and an increase in spontaneous movements.

Conversely, *Stx1b* (Syntaxin 1B) and *chd2* (chromodomain helicase DNA binding protein 2) knockdown larvae presented an alteration in their seizure activity [90,91]. In this framework, a loss of function of 50% of *Stx1b,* associated with fever epilepsy syndromes, caused an abnormal behavior in ZF larvae characterized by the absence of response to touch at 4 dpf and unusual and low-frequency convulsions at 5 dpf [91]. Mutations in the human *CH2* gene are involved in a wide range of epileptic encephalopathies, including myoclonic–atonic epilepsy, Dravet syndrome, and Lennox–Gaustat syndrome. Morphant larvae presented different malformations, including body curvature, pericardial edema, and a smaller head in comparison to control samples [90]. Moreover, they displayed abnormal behavior with whole-body trembling, pectoral fin, jaw twitching, and some epileptiform discharges [90]. The duration and frequency of discharges were longer and higher, respectively than in the controls [77]. Of notice, these parameters were increased in light conditions. These results confirm that mutations in the *CH2* gene are involved in photosensitivity-induced seizures.

As said, a variety of models of monogenic epilepsy have been produced by profiting from ZF and a complete list of them is presented in Table 1.

**Table 1:** Genetic zebrafish epilepsy model.

Human Gene	ZF Correspondant Gene	Protein Encoded	Name Mutants/Morphants	Syndrome Associated	Main Characteristics	Reference
MIB1	*mind bomb*	E3 ubiquitin ligase	mib^hi904^ mutants	Angelman syndrome	Locomotor activity consistent with Stages I and III; high spike bursts.	[88]
LGI1	*lgi1a*	Secreted protein involved in protein–protein interactions	*lgi1a* morphants	ADPEAF or ADLTE	Smaller eyes and brain compared to the controls; tail malformations; hyperactivity with seizure behavior; PTZ exposure: increase in activity.	[89]
KCNQ3	*Kcnq3*	K_v_7.3 potassium channel	*Kcnq3* morphants	BFNC	LPD treatment: significant increase in locomotor activity.Low dose of LPD: Stage I High doses of LPD: Stage III.	[87]
SCN1A	*Scn1Lab*	α subunit of Na_V_1.1, a voltage-gated sodium channel	Didy^s552^	DS	Increase in their locomotor activities and seizures, classifiable as Stage III: activity similar to the one described in larvae treated with PTZ; Exposure to stiripentol, PB, DZP, and VPA: decrease in seizures.	[82]
SCN1A	*Scn1Lab*	α subunit of Na_V_1.1, a voltage-gated sodium channel	*Scn1Lab*	DS	Whole-body seizures.	[83]
SCN1A	*Scn1Lab*	α subunit of Na_V_1.1, a voltage-gated sodium channel	*Scn1Lab*	DS	Anomalies in the body axis; hyperpigmentation; increase in the number of movements between 3 and 5 dpf, with abnormal hyperactive locomotor activity; decrease seizures after exposure to anticonvulsant.	[84]
SCN1A	*Scn1Lab*	α subunit of Na_V_1.1, a voltage-gated sodium channel	*Scn1Lab*	DS	GABAergic neuronal loss.	[92]
CH2	*Ch2*	Chromodomain helicase DNA-binding protein 2	*Ch2* morphants	Epileptic encephalopathies (myoclonic–atonic epilepsy, Lennox-Gaustat, and DS)	Perturbation in the locomotor activity; seizure behavior.	[90]
CH2	*Ch2*	Chromodomain helicase DNA-binding protein 2	*Ch2* morphants	Epileptic encephalopathies (myoclonic–atonic epilepsy, Lennox-Gaustat, and DS)	Photosensitivity-induced seizures.	[93]
KCNJ10	*kcnj10a and kcnj10b*	Inward-rectifying potassium channel Kir4.1	*Kcnj10a* morphants	EAST/SeSAME syndrome	Paroxysmal events; Exposure to DZP: no effects; Exposure to PBB: reduction of events.	[86]
KCNJ10	*kcnj10a*	Inward-rectifying potassium channel Kir4.1	*Kcnj10a* morphants	EAST/SeSAME syndrome	At 30 hpf high number of body contractions; at 5 dpf abnormal swimming and posture.	[85]
STX1B	*stx1b*	Plasma membrane synaptic protein Syntaxin-1B	*stx1b* morphants	Fever-associated epilepsy syndromes	40% of larvae: no touch response at 4 dpf; starting from 5 dpf unusual and low convulsive behavior, including myoclonus-like jerks.	[91]
ALDH7A1	*aldh7a1*	Enzyme α-aminoadipic-semialdehyde-dehydrogenase	*aldh7a1^−/−^*	PDE	Abnormalities in body axis; hyperactivity and seizure behavior.	[94,95]
DEPDC5	*depdc5*	DEP domain-containing protein 5	*depdc5^−/−^*	Autosomal-dominant familial focal epilepsy	At 17 hpf hyperactivity; at 48 hpf abnormal motor activity.	[96]
DEPDC5	*depdc5*	DEP domain-containing protein 5	*depdc5^−/−^*	Autosomal-dominant familial focal epilepsy	Larvae shorter in body length compared to control; reduction of locomotor activity, that increases after PTZ exposure.	[97]
GABRA1A	*gabra1*	α1 subunit of the GABA A receptor	*gabra1^−/−^* mutants	Generalized epilepsy	Epileptic phenotype; a decrease of the convulsions after exposure to the tonic–clonic-like seizures, which were abolished by prior exposure to LVT, VPA, and CLZ.	[98]
PLPBP	*plpbp*	PLP homeostasis protein	*pbp^−/−^* mutants	PDE	Epileptic phenotype.	[99]
CACNA1A	*cacna1a*	α1 subunit of P/Q calcium channels	*cacna1aa* morphants	Early-onset epileptic encephalopathy, infantile epileptic, infantile epilepsy with myoclonus and febrile seizures	Shorter body compared to controls; hyperpigmentation; epileptic phenotype.	[100]
GABRG2	*gabrg2*	GABAA receptor gamma 2 subunit gene	GABRG2F^343L^ mutants	EOEEs	Seizures behavior; hyperactivity; After treatment with SAHA: dose-dependent decrease in swimming distance; After exposure to VPA, CLZ, LVT, and CBZ: no changes.	[101]

Abbreviations: ADLTE: autosomal dominant lateral temporal lobe epilepsy; ADPEAF: autosomal dominant partial epilepsy with auditory auras; ALDH7A1:aldehyde dehydrogenase 7A1; BFNC: Benign familial neonatal convulsion; CACNA1A: calcium voltage_gated channel subunit alpha 1A; CBZ: carbamazepine; CH2: chromodomain helicase DNA binding protein 2; CLZ: clonazepam; DEPDC5: DEP domain-containing protein 5; dpf: days postfertilization; DS: Dravet syndrome; DZP: diazepam; EAST/SeSAME: epilepsy, ataxia, sensorineural deafness, and renal tubulopathy; EOEEs: Early onset epileptic encephalopathies; GABRA1A:gamma-aminobutyric acid receptor subunit alpha-1; hpf: hours postfertilization; KCNJ10: ATP-sensitive inward rectifier potassium channel 10; KCNQ3: potassium voltage-gated channel subfamily KQT member 3; LGI1: Leucine-rich, Glioma-Inactivated 1; LPD: linopirdine; LVT: levetiracetam; MIB1: MIB E3 Ubiquitin protein ligase 1; PB: potassium bromide; PBB: pentobarbitone; PDE: Pyridoxine-dependent epilepsy; PLPBP: PLP homeostasis protein; PTZ: pentylenetetrazole; SAHA: suberanilohydroxamic acid; SCN1A: sodium voltage-gated channel alpha subunit 1; STX1B: syntaxin 1B; VPA: valproic acid.

### 4.4. Other Types of Epilepsy Models in ZF

Apart from the classical approaches above described, there are a variety of models used in ZF which have explored interesting aspects of human epilepsy. For instance, the aspect of the effect of external temperature on seizure threshold has been assessed in adult ZF, which were exposed to water temperature up to 30°, showing a higher proneness to seizures by PTZ in parallel with a modified effect of pretreatment with glutamatergic receptor antagonists on those seizures [102]. Very recently, hyperthermia has been used to reduce seizure threshold to PTZ also in larval ZF, and it has been shown that this is associated with the production of proinflammatory cytokines in line with what is hypothesized to occur in human febrile seizures [103].

Another interesting protocol for seizure induction in adult ZF was recently developed by Kumari et al., consisting in inducing chronic epileptogenesis through repeated exposure to subthreshold concentrations of PTZ, in what was considered a chemical kindling model by the authors themselves [104]. In particular, adult ZF were daily exposed to freshly prepared 1.25 mM PTZ for 30 min. Regular PTZ exposure was stopped until a significant number of fish showed a repeated score of five or above seizures, thrice, and were considered as kindled. In this study, a seven-stage seizure score was used resembling the one used for Kainic acid. More than 65% of fish experienced were kindled after 22 days. In these kindled ZF, it was analyzed, not only the tissue content of different neurotransmitters but also their cognitive outcome and it was observed that PTZ kindling induced spatial cognition deficits and lower social exploration in ZF [105].

On a similar line, PTZ kindling followed by a single dose of KA [106] has been recently used as a “kindling-like” model of seizures in adult ZF: in particular, it was shown that daily injection of PTZ at 80 mg/kg daily, for 10 consecutive days makes a final injection of KA 3 mg/kg convulsant, while KA alone, in non-PTZ-kindled ZF, did not induce seizures. In the same study, Kundap et al. could demonstrate also that the PTZ and KA groups showed memory impairment in an ad hoc developed three-axis maze [106].

Finally, and quite unexpectedly, in the last three years, several studies have addressed the use of ZF to test post-traumatic epilepsy (PTE), a frequent cause of symptomatic epilepsy in humans. In particular, after the first report by Cho et al. (2020) [48] who used controlled high-intensity focused ultrasound to induce different degrees of traumatic brain injury, and showed the appearance of both spontaneous seizures and lowered threshold to PTZ in 100% of animals, four more studies were published in 2021 confirming the usefulness of ZF to model PTE [107,108,109,110,111]. This model has been further detailed in terms of the degree and site of traumatic insult required to produce the PTE [48,107,108]. An example of its potential is represented, for instance, by the fact that through its application, and profiting from the relatively easy feasibility of ZF with a genetically encoded fluorescent Tau biosensor (reporting in vivo accumulation of human Tau species), it has been recently proposed that there is a link between traumatic brain injury, subsequent tauopathy, and PTE [111].

## 5. The Tools for Assessing Seizures in ZF

In the previous paragraphs, we mentioned the seizure models for which ZF have been used up to now. The approaches through which seizures can be assessed vary from model to model and among different laboratories. The monitoring of seizures is one of the key points for the precise epileptic phenotype identification and assessment of specific treatments. The gold standard for identifying seizures is, by definition, also in humans, represented by the EEG recording of epileptic spikes and electrographic seizures; however, due to technical issues, and the need for immobilizing the animals for EEG recordings, behavioral motor scores are mostly used for seizures analysis in ZF.

Concerning the electrophysiological recordings of spikes, the approaches for the analysis of seizures in ZF models are novel, evolving, and emerging. In this framework, different approaches ranging from the single patched cell to an electroencephalogram of the entire brain have been developed, used, and optimized. The currently available in vivo technologies to observe and monitor the electrographic seizure events in ZF with chemically-induced seizures or in ZF epilepsy models (epileptic morphant/transgenic fish) consist generally of video tracking approaches to assess changes in the neurobehavioral profile of ZF (swimming activities, spontaneous movements, and touch response), as already described [31,86], as well as sophisticated and invasive tools [31,112,113], microfluidic systems [114,115], and different fluorescence microscopy techniques to monitor the electrographic events [116,117,118].

### 5.1. Electrophysiological Techniques

Electrographic recordings provide very high temporal resolution and enable the comparison with the results obtained during electrophysiological measurements in mice, rats, and humans. In particular, the monitoring of the local field potential in ZF brains has been widely performed to investigate the possible electrographic effects or changes in ZF treated with different drugs and convulsants [31,63,82,88,119]. Even if these kinds of measurements are extensively employed in ZF, the precise electrographic characteristics of electrographic seizures in these organisms after treatment with different compounds/drugs are little known [120]. These electrographic measurements are often applied to larvae. Zebrafish larvae between 3 and 7 dpf are usually embedded in 1.2–2% low-melting-point agarose prepared in an embryo medium. One or two glass microelectrodes filled with NaCl (2 M) or with artificial cerebrospinal fluid (124 mM NaCl, 10 mM glucose, 2 mM KCl, 2 mM MgSO_4_, 2 mM CaCl_2_, 1.25 mM KH_2_PO_4_, and 26 mM NaHCO_3_ NaCl) are inserted through the epidermis, into the intracranial space, into the midbrain or optic tectum or forebrain [31,63,77,78,82,87,88,91,92,119,120,121,122,123,124]. Then, the larvae are usually treated with different doses of chemicals/drugs, and the activity is measured for 10–30 min and the obtained seizure events are compared to one of the control samples (untreated larvae). The recordings are analyzed post hoc with the appropriate software. A threshold for detection of the spike/epileptiform is usually set at three times the background activity amplitude.

The recording of electrographic events by optical mapping takes advantage of the optical transparency of the larvae brain, allowing good and wide imaging by using transgenic lines or appropriate dyes to label specific brain regions or areas or specific neurons. This kind of measurement compared to the electrographic recordings with glass microelectrode presents different advantages. First, the imaging approaches are not invasive; moreover, the microscopy measurements provide a recording of the activity in thousand neurons or the whole brain [118], allowing for the getting of information from the single-cell behavior up to the whole-brain dynamics. Transgenic ZF with calcium indicator has been employed to monitor the neuronal activity or perform in vivo imaging of network events or fast volumetric imaging using different fluorescence techniques including simple fluorescence microscopy, fast confocal microscopy, two-photon light-sheet microscopy and spinning disk confocal microscopy [116,117,118,125,126].

Few studies reported the use of microfluidic device systems to perform neuronal electrophysiological recordings in ZF larvae [114,115]. These approaches combine the use of microfluidic channels with a needle or electrode to monitor the neuronal electrophysiological activities with low noise and high sensitivity in chemically-induced seizures of ZF [115] or genetic epilepsy models [114].

Recently, two separate studies proposed for the first time the use of microelectrode arrays (MEAs) as an innovative and noninvasive tool to record the neuronal electrophysiological activities of ZF larvae with epileptic seizures [127] or ZF larvae treated with an ASD [128]. MEAs represent one of the primary investigation methods of neuronal networks used to register extracellular field potentials [129]. In particular, commercially available MEAs with 61/64 electrodes (from MED64 System or Axion Biosystems) were employed to record the electrophysiological activities in the brain and/or spinal cord of ZF larvae treated with PTZ or potassium chloride (KCl) [127] or valproic acid [128]. The measurements of ZF larvae treated with PTZ and KCl showed a significant increase in the firing activity, both in terms of spike and burst rates, with an increasing spiking activity pattern, compared to untreated larvae [127]. On the other hand, larvae treated with valproic acid (500 µM) presented a decrease in brain activity, in parallel with no significant variations in the spinal cord one, indicating that this drug principally influences brain function [128]. These studies clearly showed that the capability of using MEAs to quickly record the electrophysiological neuronal activities of ZF larvae allows the analysis of different electrophysiological phenotypes, and in particular the chemoconvulsant-induced epileptic spikes. This capability, together with the good temporal and spatial studies resolution of this noninvasive approach, opens a new perspective on the potential future of epileptogenesis studies. In comparison to the other available approaches to detect ZF neuronal activity, MEAs represent a noninvasive and highly accurate technology that allows a fast and low-cost assessment of neuronal function. In addition, this approach is label-free since it does not require the use of fluorescent dyes or transgenic lines. This avoids the process of reagent labeling (laborious and time-consuming) and the potential introduction of artifacts that can affect or forge the results of the measurements.

### 5.2. Motor Tracking in Epilepsy Pharmacological Models

The analyses of locomotor activities are the most common tools employed to investigate, screen, and monitor seizures in ZF, either adults or larvae, at different stages of development and both in wild type and mutants/morphant.

The swimming behavior in epileptic ZF larvae is generally evaluated in terms of different parameters: the speed and the total distance of swimming, and seizure activities. Thanks to the ZF model characteristics, this behavior and activities can be easily and quickly monitored and recorded in vivo under different conditions (light/dark, control/drug-treated samples) by using a camera (CCD camera or high-resolution camera) and different video tracking/recording approaches.

The evaluation of locomotor behavior in adult ZF is performed by assessing different endpoints monitored by an operator and, as in the case of the larvae, by using different video tracking/recording approaches. The parameters evaluated manually in the tank of adults included traditional endpoints, such as the number of erratic behaviors, number of transitions to the upper part of the tank, time spent in the top/upper half of the tank, and specific seizures endpoints, including circular swimming, hyperactivity and spams of hyperactivity, spasms, corkscrew swimming, and circular swimming [67,117]. The parameters monitored, and calculated by video tools include the average speed, the swimming pattern, and the total distance of swimming.

The behavioral analysis to study seizure was employed for the first time by Baraban’s group in 2005 when they developed and described a PTZ-induced seizure model in ZF larvae [31]. ZF larvae treated with different doses of PTZ first showed a significant increase in their locomotor activity (which they classified as Stage I), with subsequent fast “whirlpool-like” activity (Stage II), and short clonic-like convulsions (Stage III) causing immobility and “posture loss” for a couple of seconds [31]. As expected in the case of seizure-related behavior, the above-mentioned motor behavior of PTZ-exposed larvae was shown to be dose-dependent. In particular, larvae treated with a low dose of PTZ presented only an increase in the swimming activity and “whirlpool-like” activities, and most of them showed also one convulsion at the highest-tested dose. A deeper analysis confirmed a seizure-like behavior with a significant increase in both the number of movements and distance of swimming compared to controls [1]. Subsequently, several studies have extensively validated PTZ-induced seizures in ZF as a reproducible experimental model, showing also the similarity of the behavior shown in this model with the rodent one [31,121]. As already mentioned, most of these studies have been widely employed to screen and evaluate the effect of several compounds or drugs allowing them to identify their antiseizure/antiepileptic effects [87,119,122,130,131] (Table 2). Generally, ZF between 3 and 7 dpf were treated with a specific dose of PTZ diluted in an embryo medium, before or after treatment with the compound or drug under investigation. Alteration in the motor activities, and specifically seizure activities, are visible after seconds/minutes of observation. A concentration of PTZ of 15 or 20 mM has been indicated as the dose appropriate to induce an increase in swimming behavior [31,121,124]. A recent study has optimized the protocol for PTZ ZF developed by Baraban, analyzing a range of convulsant doses between 5 and 20 mM on larvae at the same stage of development (7 dpf) [131].

Few studies have reported the changes in locomotor activity of larvae treated with different doses of KA. During the development, the monitoring of seizure characteristics was poorly evaluated even with electrographic recordings coupled with a reduction of neuronal cell proliferation [49,51]. One study investigated both the impact of different concentrations of KA on the motor activity of ZF in the early stages and the impact of the pretreatment with KA in juvenile animals [120]. ZF treated with KA exhibited different behavior depending on the stage of development analyzed. In particular, larvae treated at an early stage with KA (100, 300, and 500 μM) presented a decrease in their motor activity at 7 dpf with a reduction in the distance of swimming in comparison with the control samples. At 15 dpf, the swimming activity increased in larvae exposed to the highest concentration of KA tested, while at 30 dpf, no effects were observed. However, the treated larvae did not present a seizure behavior [120]. An analysis of the seizures was performed by using a classification score of locomotor behavior defined by Alfaro et al. [49]. Based on this method, the animal can present seven different behavioral stages, with Stage I characterized by signs of immobility and hyperventilation and Stage VII by death. The larvae treated with KA did not exhibit any of the events based on this classification [120].

PTZ-induced motor behavior has been described also in adult ZF, after either bath exposure or intraperitoneal injection of the chemoconvulsant [58,67,117,118,119,120]. In this framework, two motor behavior stage classifications, differing from the abovementioned Baraban’s one, were developed and used in the same year by two different research groups. On one side, the group of Mussulini et al. monitored the behavior of adults treated with different doses of PTZ and classified them into six different stages, from short swimming (score zero), up to death (score six) [58]. Zebrafishes treated with different doses of PTZ presented different convulsive behavior. In particular, only the ZF treated with the highest concentration of PTZ exhibited a prolonged time to come back to the starting behavior. The induction of seizure was dose-dependent, with reduced motility at 5 mM of PTZ and marked hyperactivity at 15 mM. Finally, seizure severity was significantly reduced by the preadministration of diazepam (DPZ). In any case, the behavioral profile of adults treated with PTZ showed a locomotor activity similar to the one observed in larvae [58]. Another group analyzed seizures after PTZ in adult ZF by a classification characterized by seven different stages [117]. In this case, ZF were injected with 220 mg/Kg in a study assessing the anticonvulsant effect of oral gabapentin (GBP). First, adult fish were treated in different concentrations of GBP. Zebrafishes treated with PTZ exhibited a significant increase in the distance and speed of swimming compared to controls, and this was inhibited by GBT in a concentration-dependent manner [4]. In this model, ZF exposed to PTZ also showed an increase in the ZF complete turns (360°), which were reduced by the highest tested doses of GBP.

As for the larvae, the motor behavior of adult ZF is the outcome measure used by almost all of the studies in which the PTZ-ZF model has been used to assess the efficiency of commonly used ASDs or molecules with potential antiseizure properties. These are detailed in Table 2.

Recently, a modification in the PTZ ZF model was performed by treatment with the convulsant from 5 to 7 dpf daily [121].

Few studies reported the changes in locomotor activity of adults treated with different doses of KA. KA administration in adult ZF induces a sequence of motor behaviors that have been detailed by Alfaro et al. and confirmed by several authors afterward (e.g., [51]). In particular, the swimming behaviors of ZF treated with different doses of KA were monitored and classified in different stages, as in the case of larvae [50,122]. The behaviors of exposed ZF were dependent on the dose injected into the animals. Animals treated with a low concentration of KA presented behavior classifiable as stage I- IV (Stage I: immobility and hyperventilation; Stage II: whirlpool-like swimming behavior; Stage III: rapid movements from right to left; Stage IV: abnormal and spasmodic muscular contractions), while ZF injected with high concentration exhibited activities of Stage V, characterized by rapid, whole-body, clonus-like convulsions [50]. A deep study reported the locomotor activity of ZF treated with KA also in terms of speed and the total distance of swimming and time of immobility analyzed over time (until 168 h). Injected ZF exhibited a lower speed and distance of swimming between 24 and 72 h from the administration and started to present parameters comparable to the control after 96 h. All the treated ZF showed at least one behavior classifiable as Stage V [51].

## 6. Which Questions Are and May Be Asked to the ZF Model Concerning Epilepsy?

In the previous paragraphs, it was emphasized that ZF seizure models have been used mainly for screening the pro-/anticonvulsant effects of experimental or well-known molecules, and this has been performed almost exclusively in adult ZF exposed to PTZ. This remains probably the most useful application of ZF to this research field since this allows testing a large number of drugs in a limited period, in a large number of animals, and with a significant reduction of ethical constraints and costs, compared with classical animal models.

**Figure 3:**
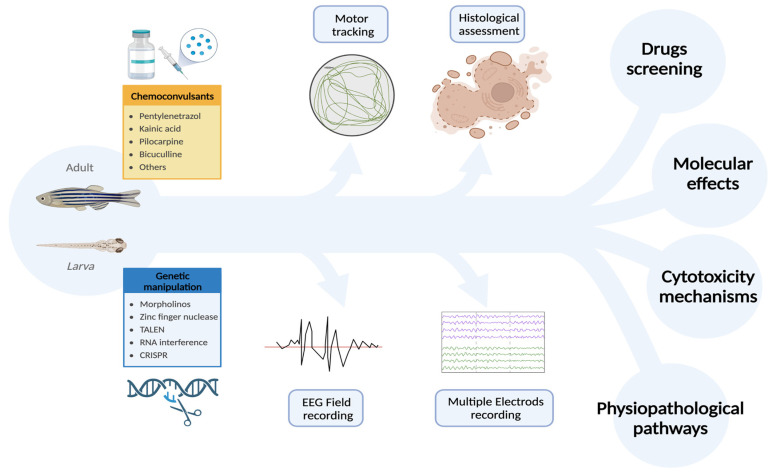
Zebrafish is an excellent animal model for studying epilepsy due to its ease of handling and accessibility. In addition to requiring fewer ethical considerations, ZF also exhibits rapid growth and efficient replication. Furthermore, ZF are sensitive to the same chemoconvulsants used in other animal models and are particularly amenable to genetic manipulations. This makes ZF a versatile experimental platform where pharmacological and genetic approaches can be successfully integrated, resulting in significant cost and time savings compared to other animal models. Importantly, the quality and quantity of assessments are not compromised, as electrophysiological and behavioral analyses can be used to accurately evaluate the outcomes of manipulations. These unique features make ZF an ideal model for investigating the pathophysiology and cytotoxic mechanisms of epilepsy, exploring the pathological role of gene mutations, and screening new antiseizure drugs. [created with Biorender.com; accessed on 3 April 2023]. Abbreviations: TALEN: Transcription activator-like effector nuclease; CRISPR: Clustered regularly interspaced short palindromic repeats of genetic information; ZF: zebrafish.

Another aspect in which ZF can provide massive pieces of information is the large-scale analysis of mutations observed in patients with epilepsy, by introducing these mutations in ZF (both adult and larvae) and analyzing seizure onset/proneness and phenotype. With this respect, a significant improvement might be provided by electrophysiological tools, including MEAs, which can reveal even subtle seizures in the subparts of the CNS (Figure 3).

More complex protocols, aimed at reproducing epileptogenesis processes have been recently developed, showing that the brain of ZF can be submitted to chemical kindling. This might offer interesting scenarios, not only in helping develop potential antiepileptogenesis drugs but also in helping to disclose the potential cellular and molecular mechanisms of epileptogenesis itself to be verified in higher mammals. In line with this, the “two-hit” model of SRS, which is widely used in mice treated with KA, has been recently shown also in ZF in a study with KA [66], in which ZF larvae experienced SRS days after exposure to KA, and it might be further extended to the other. Nevertheless, it is also worth mentioning that in rodents, the real usefulness of this approach has been challenged recently. Conversely, the epileptogenesis process occurring during PTE development has been reproduced successfully very recently in ZF, and this appears as a very promising field of research.

There are some potential aspects related to epilepsy that might be explored in the future in ZF. The neuropathological and cellular effects of prolonged seizures might be assessed in the different parts of ZF CNS, which can be easily and quickly fully assessed; this can be used as a setup on which to test neuroprotective treatments of the effects of SE. This has been, at least in part, shown in a few studies profiting from sophisticated techniques for seizure monitoring, which provide imaging of the seizure onset site in the brains of ZF with a high time and space resolution [117,123,150]; the use of MEAs with high electrode density might allow a more detailed and wider use of this subregional analysis. Moreover, also in the ZF brain, several subcortical nuclei releasing monoamines or Ach, thus resembling the locus coeruleus, raphe nuclei, and cholinergic brainstem nuclei of mammals, have been described [151,152,153,154]. All these structures have been suggested to modulate seizures in mammals [155,156,157,158], and it would be very interesting to assess their role on seizure proneness and seizure effects also in ZF, to better delineate their potential role in human epilepsy.

Finally, ZF might play a key role also in the investigation of the bidirectional link existing between epilepsy and sleep. Approximately 10–15% of all epilepsies are related to sleep, including sleep-related hypermotor epilepsy; this aspect of the epilepsy research field has been growing in the last decades and is becoming quite important. Briefly, pathologic high-frequency oscillations and interictal epileptiform discharges are frequent during nonrapid eye movement (NREM) sleep and may contribute to sleep–wake complaints [159]. Vice versa, sleep–wake disorders are more common in adults with epilepsy and can impact seizure control and quality of life [160]. Circadian biology is an emerging area of epilepsy research, as over 90% of people with epilepsy has seizures with circadian periodicity [161]. Understanding these bidirectional relationships is important for optimizing epilepsy outcomes and patient and caregiver education [162].

Given the well-established role of sleep in epileptogenesis and the modulation of brain excitability, ZF could play a role as an animal model also in this respect. In the last decades, ZF have been used also as a model organism to study the neural and genetic mechanisms of sleep [163]. While ZF do exhibit a period of reduced activity and increased rest, their sleep patterns differ from those observed in mammals. ZF do not have a clear distinction between deep and light sleep, and they can enter this resting state at any time of the day or night. Despite these differences, studies have shown that ZF show slow bursting sleep and propagating wave sleep, sharing commonalities with those of mammals’ slow-wave sleep and paradoxical or rapid eye movement sleep [164]. Further research has also shown that ZF have homologs of genes involved in regulating sleep in mammals, including hypocretin and melatonin [165]. Overall, ZF represent valuable model organisms for studying the neural and genetic mechanisms of sleep and may provide insights into sleep regulation and disorders in humans.

## 7. Conclusive Remarks

Experimental research in epilepsy represents a neuroscience field of investigation that offers the opportunity not only to help develop new potential treatments for the human disease, but also to disclose cellular and network mechanisms involved in neuronal excitability, and indirect information on brain physiology. A variety of new techniques emerged in the last several years, offering the chance to assess, in simplified organisms, in vivo and ex vivo, at macro- and microscale levels, parameters involved both in the onset and in the effect of epilepsy. An - in-depth knowledge of the comparative anatomy of these organisms allows for avoiding the overinterpretation of these results concerning the human disorder to the model. Zebrafish are a classic example of what was mentioned above, and in the future, they might offer a very useful tool for analyzing a large amount of data in a much shorter period and at lower costs compared to classical rodent models, without losing too much potentially relevant information. Obviously, the data obtained in ZF should be interpreted in the context of wider epilepsy research platforms including in vitro and in vivo studies used in other animal species and noninvasive assessments in humans. ZF larvae and adults are promising and excellent systems for epilepsy research with distinct and different peculiarities that should be considered during future epilepsy studies, including the different advantages and limitations that both stages present. As described above, ZF larvae are suitable for high-throughput screening and are easy to be handled in different experiments but do not give the possibility to study the sex differences. In addition, the incomplete neuronal system of larvae, including a BBB that is still in development, can limit epilepsy characteristics, leading to different results in some drug assessments. On the other side, the adults are more suitable for specific experimental approaches, including drug injection, EEG, and dissection, due to their size. Moreover, the completely developed and complex neuronal system of adults provides several advantages in comparison to larvae both in pharmacological and physiopathological epilepsy investigations.

## Figures and Tables

**Table 2:** Effects on the locomotor activities of the PTZ zebrafish model.

Stage	Dpf/Months	Tested Drugs/Compounds	Tested Concentrations	Locomotor Activity	Reference
Embryos	7 dpf	PTZ	2.5–15 mM	Increase in locomotor activity (Stage I), with a subsequent quick “whirlpool-like” activity (Stage II), and short clonic-like convulsions (Stage III); behavior dose-dependent.	[31]
Embryos	7 dpf	PTZ	15 mM	Mutant (s334) seizure-resistant: decrease in swimming activity; behavior classifiable as Stage II.	[63]
Embryos	6 dpf	PTZ PTZ + ETS PTZ + VPA PTZ + GBP PTZ + LTG PTZ + OXC PTZ + PMD PTZ + CBZ PTZ + PHT PTZ + LVT PTZ + TPR PTZ + TGB PTZ + DZP PTZ + ASP	PTZ: 20 mM; ETS: 3, 10, 30 mM; VPA: 300 µM, 1 µM, 3 mM; GBP: 16, 50, 160 mM; LTG: 30, 100, 300 µM; OXC: 250 µM, 790 µM, 2.5 mM; PMD: 750 µM, 2.4 and 7.5 mM; CBZ: 30, 100, 300 µM; PHT: 100, 300 µM, and 1 mM; LVT: 10, 30, 100 mM; TPR: 1, 3, and 10 mM; TGB: 30, 100, and 300 µM; DZP: 5, 16, and 50 µM; ASP: 30, 100, 300 mM.	PTZ treatment: an increase in the total distance of swimming; PTZ + one of the anticonvulsant drug: reduction of the seizures: these were observed for all the selected drugs apart for PMD.	[132]
Embryos	4 dpf	PTZ PTZ + VPA PTZ + EE PTZ + AP PTZ + NIMO PTZ + NITRE PTZ + methiothepin PTZ + pimozide PTZ + sulconazole PTZ +suloctidil PTZ + nerolidol PTZ + dioxybenzonePTZ + hexylresorcinol PTZ + retinyl acetate	PTZ: 1.25, 2.5, 5, 10, 20, 40 and 80 mM; PTZ (20 mM) + VPA: 1 mM; PTZ (20 mM) + cmpd: 0.9, 2.7, 8.2, 24.7, and 74.1 µM.	PTZ: increases in locomotor activity dose-dependent; AP, EE, NIMO, NITRE Methiothepin, pimozide, and VPA: inhibition of locomotor activity; Retinyl acetate: no effects.	[133]
Embryos	7 dpf	PTZ CUR + PTZ TO + PTZ Ar-turmerone + PTZ α,β-turmerone + PTZ α-atlantone +PTZ VPA + PTZ	PTZ: 20 mM CUR: 2.5, 5, and 10 µg/mL; TO: 2.5, 5, and 10 µg/mL; Ar-turmerone: 11, 23 and 46 µM; α,β-turmerone: 5, 11 and 23 µM; α-atlantone: 11, 23 and 46 µM; VPA: 250, 500 µM and 1 mM.	CUR or TO without PTZ: slow increase in motor activity; CUR: at all the tested doses anticonvulsant activity; TO: at 10 μg/mL reduction of seizures; Ar-turmerone: at 46 µM anticonvulant activity; α,β-turmerone: at 23 µM anticonvulant activity; α-atlantone: at 23 and 46 µM anticonvulant activity; VPA: important activity at 250, 500 µM, and 1 mM.	[122]
Embryos		PTZ ASP + PTZ CBZ + PTZ ETS + PTZ OXC + PTZ LTG + PTZ LVT + PTZ ZSM + PTZ PMD + PTZ TPR + PTZ DZP +PTZ GBP +PTZ TGB +PTZ VPA +PTZ PHT +PTZ	PTZ: 20 mM: ASP: 50 µM; CBZ: 100 µM; ETS: 10 mM; OXC: 250 µM; LTG: 100 µM; LVT: 10 mM; ZSM 300 µM; PMD: 750 µM; TPR: 200 µM; DZP: 16 µM; GBP: 25 mM; TGB: 100 µM; VPA: 1 mM; PHT: 100 µM.	DZP, TPR, VPA, GBP, and ZSM: a significant decrease in the seizures induced by PTZ; ETS, LVT, and OXC: slight decrease in the seizures induced by PTZ; CBZ, LTG, PMD, TGB, PHT: no effects.	[121]
Embryos	3 dpf	TSN IIA + PTZ	TSN IIA: 100 mM; PTZ: 20 mM.	Important reduction of movements.	[134]
Embryos	6/7 dpf	Cmpd 1 + PTZ Cmpd 2 + PTZ Cmpd 3 + PTZ Cmpd 4 + PTZ Cmpd 5 + PTZ Cmpd 6 + PTZ Cmpd 7 + PTZ Cmpd 8 + PTZ	Cmpd: 70, 140, 280 µM; Cmpd 7: 70 and 140 µM; Cmpd 8: 0.3, 3, 30 nM; PTZ: 20 mM.	Cmpd 1: at 70 and 140 µM reduction of PTZ seizures; Cmpd 2 and 3: at 70 µM reduction of seizures; Cmpd 4, 5, and 6: no effects; Cmpd 7: important reduction of PTZ effects; Cmpd 8: no significant effects.	[130]
Embryos	6 dpf	PTZ PTZ + PS PTZ + Cmpd 15 PTZ + Cmpd 17 PTZ + Cmpd 18	PTZ: 10 mM; PS: 300 µM; Cmpd 15: 30, 100, and 300 µM; Cmpd 17: 30, 100, and 200 µM; Cmpd 18: 30, 100, and 300 µM.	The tested compound showed important effects against epilepsy. PS: at 300 µM therapeutic efficiency of 63–66%; Cmpd 15: at 300 µM therapeutic efficiency of 55%; Cmpd 17: at 100 and 200 µM therapeutic efficiency of 77 and 90% respectively; Cmpd 18: at 100 and 200 µM: therapeutic efficiency of 65 and 53% respectively.	[135]
Embryos/Juvenile/ Adults	7 dpf 45 dpf 6–8 months	RAP +PTZ PTZ VPA	RAP: Larvae 0.12, 0.25, 0.5, 1, and 2.5 μM; Juvenile and adults: 0.25, 0.50, 1, 2.5, and 5 mg/kg; PTZ: 7.5 mM; VPA: larvae 3 mM; Juvenile and adults: 100 mg/Kg.	RAP + PTZ: no changes in the motor activity, latency to reach Stage III prolonged in embryos, juvenile and adult zebrafish.	[136]
Embryos	7 dpf	PTZ SC-236 + PTZ SC-560+ PTZ	PTZ: 15 mM; SC-236: 5 μM; SC-560: 2.8 μM.	SC-236 and 560: inhibition of PTZ effect; low speed and distance.	[137]
Embryos	7 dpf	PTZ	PTZ: 2.5, 5, 7.5, 10, 12.5, 15, 17.5, 20 mM.	PTZ: up to 10 mM total distance of swimming dose-dependent linearly; >10 mM: total distance of swimming dose-dependent in a quadratic manner; 5 mM: increase in motor activity that became constant after 30 min; 10 mM: gradual increase in motor activity after 15 min; 15 mM: fast increase in activity. 20 mM: fast progress to Stage II.	[131]
Embryos	5 dpf	PTZ PTZ + NE-VPA PTZ + VPA	PTZ: 1.25, 2.5, 5 and 10 mM; NE-VPA: 25, 50 and 100 µM; VPA: 25, 50 and 100 μM.	PTZ: dose-dependent locomotor activity; at 2.5, 5, and 10 mM convulsion-like behavior, involuntary, fast, and circular movements; VPA: decrease in epileptiform behavior and reduction of locomotor activity; NP-VPA: decrease in epileptiform behavior and reduction of locomotor activity more significant than for VPA alone.	[138]
Embryos	7 dpf	PTZ PTZ + NRG PTZ + KFL PTZ + NRG-M PTZ + NRG-DM PTZ + KFD	PTZ: 40 mM; NRG: 12, 25, and 50 μM; KFL: 6.25, 12,5, and 25 μM; NRG-M: 6.25, 12.5, and 25 μM; NRG-DM: 6.25, 12,5, and 25 μM; KFD: 6.25, 12.5, and 25 μM.	PTZ: increase in convulsions and motor activity; NRG: seizures decrease; KFL: seizures decrease; NRG-M: seizures decrease by 20–30%; NRG-DM: seizures decrease by 20–30%.	[119]
Embryos	7 dpf	SUL DPH Yohimbine MK-801 VPA PTZ	SUL: 4, 20, 100, 500, and 1000 μM; DPH: 1, 4, 20, 100, 500 μM; Yohimbine: 10, 25, 50, 100, and 200 mg/L; MK-801: 10, 20, 50, 100 and 200 μM; VPA: 1, 4, 20, 100, 500 μM; PTZ: 1, 2, 4, 8 and 16 mM.	SUL: no effects in dark or light conditions; at 500 μM important increase in the lighting motor index; DPH: at 500 μM important decrease in motor activities; Yohimbine: at medium and high doses (25, 50, 100, and 200 mg/L) decrease in locomotor activity in light conditions; at 10 mg/L increase in movements, while at the other doses important reduction in dark conditions. MK-801 light condition: dose-dependent decrease in the total distance of swimming; dark conditions dose-dependent downward behavior; VPA: light and dark conditions: at 500 μM important decrease in motor activities; PTZ: light conditions: at 1–8 mM increase in motor activity; at 16 mM behavior comparable to the control samples; dark conditions: at 16 mM important decrease in activity.	[139]
Embryos	3 dpf	PTZ	PTZ: 5 mM	Hyperactivity.	[140]
Embryos	121 hpf	PTZ CBZ + PTZ LVT +PTZ LTG +PTZ PB + PTZ PHT + PTZ VPA + PTZ	PTZ: 2.5 mM; For all the antiepileptic drugs: 6.25, 12.5, 25, 50, or 100 μM.	PHT and LTG: at 50 and 100 μM important decrease of reduced epileptiform behavior; VPA: at 100 μM anticonvulsant effect; CBZ, LEV, and PB: no effects.	[141]
Embryos/Adults	7 dpf 3 month old	PTZ PTZ + LY294002	Embryos: PTZ: 8 mM; LY294002: 1–50 µM; Adults: PTZ: 6 mM; LY294002: 10–400 mM.	Embryos: a significant decrease in mean speed and the total distance of swimming; an increase in latency to seizures. Adults: decrease in seizures, increase in latency to seizures.	[142]
Embryos	117 hpf	PTZ PTZ + PTE	PTE: 10 μM; PTZ 20 mM.	PTZ led to an important reduction of PTZ-induced zebrafish activity.	[124]
Embryos	5 dpf	PTZ PTX TETS	PTZ: 0.1–100 mM; PTX: 0.01–4 mM; TETS: 0.1–100 μM.	PTZ: seizures increase dose-dependent (score 3 the highest dose); PTX: seizures increase dose-dependent (score 3 at the highest dose); TETS: seizures increase not dose-dependent.	[79]
Embryos	7 dpf	PTZ SS	PTZ: 15 mM; SS: 100 μM, 200 μM, 400 μM.	SS: important reduction of seizures induced by PTZ (with final suppression) and reduction of total distance and speed of swimming.	[143]
Embryos	6/7 dpf	PTZ PTZ + halimide PTZ+ plinabulin	PTZ: 40 mM; Halimide: 50, 100 and 200 μg/mL; Plinabulin: 1.25, 2.5, 5 and 10 μM.	Halimide: reduction of seizures; Plinabulin: important decrease of seizures.	[77]
Embryos	4 dpf	PTZ VPA	PTZ: 10, 20 and 40 mM; VPA: 1, 3 and 5 mM + PTZ (10 mM).	PTZ: 10 and 20 mM important increase in movements; 40 mM: strong increase in movements; VPA: 5 mM decrease in movements.	[144]
Adults	-	PTZ VPA + PTZ	PTZ 10 mM; VPA: 100-200 μM.	PTZ: seizure behaviors; increase in the total distance of swimming; VPA: the disappearance of Stage II; reduction of the increase of swimming induced by PTZ.	[145]
Adults	5–7 months	PCT PTZ CAF	PCT: 0.7 mM; PTZ: 11 mM; CAF: 1.3 mM.	High hyperactivity	[59]
Adults	-	PTZ GBP + PTZ	PTZ: 220 mg/Kg; GBP: 200–400 and 600 mg/Kg.	An important increase in swimming velocity prevented by the administration of the highest concentrations of GBP; high mobile frequency and duration is inhibited by the GBP administration in a dose-dependent mode; an increase in the zebrafish complete turns (360°), reduced by the highest tested doses of GBP.	[146]
Adults	4–6 months	PTZ	PTZ: 5, 7.5, 10 and 15 mM	Induction of seizure dose-dependent; at 10–15 mM mortality rate of 33 and 50% respectively; at 15 mM high hyperactivity.	[72]
Adults	4–5 months	PTZ PTZ + PGBL PTZ + LC PTZ + DZP: PTZ + GBP PTZ + CBZ PTZ + VPA PTZ + CAF	PTZ: 6 mM; PGBL: 10 nM; LCS: 1 µM–3 mM; DZP: 1–100 μM; GBP: 100 μM–10 mM; CBZ 1–100 μM; VPA 100 μM–10 mM; PTZ: 4 mM; CAF: 30 μM.	Increase in seizure latency noted in a dose-dependent manner for: −300 μM–10 mM of VPA −10–100 μM of CBZ −100–3 mM LCS −10 mM of GBP CBZ: at 100 μM absence of Stage II and II, as well as the high doses of DZP; No effects for PGBL; CAF: reduction in seizure latency.	[147]
Adults	3 months	PTZ PTZ + VPA PTZ + SC PTZ + PP	PTZ: 6 mM VPA: 300 μM; SC: 1 μM; PP: 9 μM.	VPA: reduction of hyperactivity; SC: increase of the latency to seizures and spasms, reduction of spasms and hyperactivity; PP: increase of the latency to spasms.	[148]
Embryos/Adults		CUR + PTZ Micronized CUR +PTZ VPA + PTZ	CUR: 1 μM Micronized CUR: 1 μM VPA: 3 mM CUR: 0.50 mg/kg; Micronized CUR: 0.50 mg/kg VPA: 100 mg/kg	Larvae: CUR and micronized CUR: no alteration in the swimming distance VPA: reduction of swimming distance; Adults: CUR, micronized CUR, VPA: decrease in locomotor activity;	[149]

Abbreviations: AP: Allopregnanolone; CAF: caffeine; CBZ: carbamazepine; CMPD: compound; CUR: curcuminoids; dpf: days postfertilization; DPH: diphenylhydantoin; DZP: diazepam; EE: ethinyl estradiol; ETS: ethosuximide; GBP: gabapentin; Hpf: hours postfertilization; KFD: kaempferide (40-O-methyl kaempferol); KFL: kaempferol; LCS: lacosamine: LPD: linopirdine; LTG: lamotrigine; LVT: levetiracetam; NE-VPA: nutraceutical emulsion containing valproic acid; NIMO: nimodipine; NITRE: nitrendipine; NRG: naringenin; NRG-DM: naringenin 40,7-dimethyl ether; NRG-M: naringenin 7-O-methyl ether; OXC: oxcarbazepine; PCT: picrotoxin; PGBL: pregabalin; PHT phenytoin; PMD: primidone; PTZ: pentylenetetrazole; RAP: rapamycin; SC: sodium cyclamate; SS: schaftoside; SUL: sulpiride; TETS: tetramethylenedisulfotetramine; TGB: tiagabine; TO: turmeric oil; TPR topiramate; TSNIIA: tanshinone IIA; VPA: valproic acid; ZSM: zonisamide.

## Data Availability

Not applicable.
